# Step-Wise Dual Dynamic DPSGD: Enhancing Performance on Imbalanced Medical Datasets with Differential Privacy

**DOI:** 10.3390/e28040409

**Published:** 2026-04-04

**Authors:** Xiaobo Huang, Fang Xie

**Affiliations:** Guangdong Provincial Key Laboratory of IRADS, Beijing Normal-Hong Kong Baptist University, Zhuhai 519087, China; t330026062@mail.bnbu.edu.cn

**Keywords:** differential privacy, imbalanced dataset, deep learning, convolutional neural network, dynamic differentially private mechanism

## Abstract

The application of differential privacy in deep learning often leads to significant performance degradation on class-imbalanced medical datasets. Methods such as adding noise to gradients for differential privacy are effective on large datasets, like MNIST and CIFAR-100, but perform poorly on small, imbalanced medical datasets, like HAM10000 and ISIC2019. This is because the imbalanced distribution causes the gradients from the few-shot classes to be clipped, resulting in the loss of crucial information, while the majority classes dominate the learning process. This leads the model to fall into suboptimal solutions early. To address this issue, we propose SDD-DPSGD, which uses a step-wise dynamic exponential scheduling mechanism for noise and clipping thresholds to preserve gradient information. By allocating more privacy budget and employing higher clipping thresholds during the initial training phases, the model can avoid suboptimal solutions and improve its performance. Experiments show that SDD-DPSGD outperforms comparable algorithms on the HAM10000 dataset, and the ISIC2019 dataset.

## 1. Introduction

Deep learning is widely used in sensitive areas like medicine, where datasets (e.g., patient images) are often imbalanced. This poses dual challenges: protecting privacy from known attacks [1] and ensuring fairness, as imbalanced data can lead to biased models [1].

While differential privacy (DP), typically via DPSGD, is the standard for privacy, its application to imbalanced medical datasets like HAM10000 has yielded poor performance [2,3]. Prior solutions have focused on data-centric approaches like pre-processing or class-weighting [4]. The primary limitation remains algorithmic. Existing work has not directly addressed the imbalanced problem by dynamically allocating the privacy budget based on training dynamics; while general-purpose dynamic algorithms like Auto-DPSGD-L and Auto-DPSGD-S [5] exist, they have critical flaws for this problem: they use uniform step arrangements and high-noise initialization, which are incompatible with the training dynamics of imbalanced data. Their high, automatic clipping thresholds and uniform step settings are ill-suited for datasets like HAM10000. The primary limitation remains algorithmic. Existing work has not directly addressed the imbalance problem by dynamically allocating the privacy budget based on training dynamics; while general-purpose dynamic algorithms like Auto-DPSGD-L and Auto-DPSGD-S [5] represent an outstanding approach, they exhibit critical flaws for imbalanced contexts. Specifically, they rely on uniform step arrangements and automatically default to high initial noise multipliers. This uniform scheduling is fundamentally incompatible with the fragile, low-magnitude gradients of minority classes in datasets like HAM10000.

To overcome these limitations, we introduce SDD-DPSGD. Our approach is based on a dual-schedule: exponentially increasing steps for the noise multiplier; exponentially decaying steps for the clipping threshold. This design enables a smaller noise multiplier and a reasonable clipping threshold during the critical early-training phase, protecting the fragile gradients of minority classes to avoid suboptimal solutions. Our mechanism uses non-uniform steps and leverages Rényi differential privacy (RDP) to accurately compose the total privacy budget.

### 1.1. Contributions

We evaluate our method on the HAM10000 [3] and ISIC2019 [6] datasets. Our contributions are listed below

- We propose SDD-DPSGD, a dynamic DP algorithm with two schedules: a novel, non-uniform increasing step mechanism for the noise multiplier (extending [4]) and a decaying schedule for the clipping threshold. This design is tailored for imbalanced data, starting with low noise to protect minority class gradients.
- We provide a comparative analysis of our method against DPSGD and Auto-DPSGD-S/L on the HAM10000 and ISIC2019 datasets, including an investigation of key hyperparameters (decay parameters, noise ratios, and step settings).
- We demonstrate empirically that SDD-DPSGD consistently outperforms prior methods. On HAM10000, it achieves 1.00%, 0.70%, and 0.80% higher accuracy than Auto-DPSGD-S at ϵ=3, ϵ=8, and ϵ=16 (with $\delta =10^{-3}$), respectively.

### 1.2. Notations

To enhance readability, the primary mathematical symbols used throughout this study are summarized and categorized as Table 1.

## 2. Related Work

### 2.1. Dynamic DPSGD Algorithms

Significant research has aimed to improve standard DPSGD by incorporating dynamic and adaptive mechanisms. Early work focused on adaptive learning rates [7]. Subsequent efforts primarily targeted adaptive gradient clipping, proposing methods based on validation data [8], global statistics derived from convergence properties [9] which was extended by DPAG [10], adaptive estimation via techniques like DPAdaMod-AGC [11], gradient history [12], or even per-sample thresholds [13]. Dynamically adjusting the noise multiplier, often through decay schedules, has also been explored [11,14].

Auto-DPSGD-S and Auto-DPSGD-L [5] represents an outstanding approach, combining dynamic noise multipliers (using exponential, step, or time decay) with clipping thresholds estimated from gradient norms. However, even its most effective variant (step decay) uses uniform step sizes. This uniform scheduling, coupled with challenges in robust gradient norm estimation and potentially high initial noise, is suboptimal for the non-uniform training dynamics observed in imbalanced datasets. Critically, these dynamic methods are designed for general utility, not specifically addressing class imbalance.

### 2.2. Implementation of Differential Privacy on Imbalanced Datasets

Applying DP to imbalanced datasets introduces significant fairness challenges, as DP mechanisms tend to disproportionately harm the performance on minority classes. This disparate impact is well-documented across various domains: standard DPSGD yields poor results on imbalanced medical datasets like HAM10000 [2], favors majority classes [1], and actively widens accuracy gaps during training compared to non-private models [1,15]. Similar performance degradation for underrepresented groups has been observed in facial recognition [16,17], sentiment analysis [18], and species classification [19].

Existing solutions primarily focus on data-centric methods like pre-processing, class-weighting [4], or using local DP [20]; while algorithmic fairness in DP is an active research area, with methods like calibrated functional mechanisms [21] or specific algorithms for fair binary classification [22], these do not directly address the underlying issue from the perspective of training dynamics. Specifically, they do not propose dynamic scheduling of DP parameters (noise and clipping) tailored to protect fragile minority gradients during crucial early-training phases. Recognizing this gap, and motivated by the limitations of general-purpose dynamic methods like Auto-DPSGD-S and Auto-DPSGD-L [5], our work develops a novel scheduling algorithm specifically for imbalanced data.

### 2.3. Privacy in Distributed and Resource-Constrained Environments

While our study focuses on centralized differentially private training, privacy leakage and adversarial attacks are equally critical in distributed paradigms. Recent advancements have highlighted the necessity of robust architectures in resource-constrained environments, such as stable federated learning architectures against adversarial attacks [23]. Furthermore, securing intrusion-detection systems [24] and mitigating DDoS attacks via federated learning approaches [25] demonstrate the growing need for adaptive privacy mechanisms. Incorporating differential privacy into federated learning to mitigate inference attacks via randomized response [26] shares philosophical similarities with our dynamic scheduling, as both seek to balance privacy utility under complex data distributions.

## 3. Preliminaries

### 3.1. Differential Privacy

Differential privacy is a methodology to protect sensitive information from illegal queries.

**Definition 1**  (Differential Privacy [27]). *A randomized mechanism F provides (ϵ,δ)-differential privacy if, for any two datasets D and $D^{\prime }$ that differ in only a single data point d, ∀S⊆Range(D),*

$$\mathrm{Pr}\left(F\left(D\right)\in S\right)<e^{\epsilon }\times \mathrm{Pr}\left(F\left(D^{\prime }\right)\in S\right)+\delta .$$

Usually, to implement randomness, we add Gaussian noise $N(0,\sigma^{2}\cdot s_{f}^{2})$ on a mechanism *f* with $l_{2}$ sensitivity $s_{f}$, which is introduced below.

**Definition 2**  ($l_{k}$-Sensitivity [28]). *For a function $f:X^{n}\to R^{d}$, we define its $l_{k}$ norm sensitivity (denoted as $\Delta_{k}f$) over all neighboring datasets $x,x^{\prime }\in X^{n}$ differing in a single sample as*

$$\underset{x,x^{\prime }\in X^{n}}{\sup}{∥f\left(x\right)-f\left(x^{\prime }\right)∥}_{k}\leq \Delta_{k}f.$$

In this paper, we are using $l_{2}$ sensitivity.

### 3.2. Rényi Differential Privacy

Rényi differential privacy (RDP) is defined through Rényi divergence.

**Definition 3**  (Rényi Divergence [29]). *Given two probability distributions A and $A^{\prime }$, the Rényi divergence of order α>1 is*

$$D_{\alpha }\left(A∥A^{\prime }\right)=\frac{1}{\alpha -1}\ln E_{x∼A^{\prime }}\left[{\left(\frac{A(x)}{A^{\prime }\left(x\right)}\right)}^{\alpha }\right],$$

*where $E_{x∼A^{\prime }}$ denotes the expected value of x for distribution $A^{\prime }$, and $A\left(x\right),A^{\prime }\left(x\right)$ denote densities at x.*

**Definition 4**  (Rényi Differential Privacy [30]). *For any neighboring datasets $x,x^{\prime }\in X^{n}$, a randomized mechanism $M:X^{n}\to R^{d}$ satisfies (α,R)-RDP if*

$$D_{\alpha }\left(M\left(x\right)∥M\left(x^{\prime }\right)\right)\leq R.$$

**Definition 5**  (RDP of Gaussian Mechanism [30]). *Assuming f is a real-valued function with sensitivity $s_{f}$, the Gaussian mechanism for approximating $f^{\prime }$ is*

$$f^{\prime }\left(D\right)=f\left(D\right)+N\left(0,s_{f}^{2}\sigma^{2}\right),$$

*where $N(0,\mu^{2}\sigma^{2})$ is a normally distributed random variable (standard deviation μσ, mean 0). The mechanism satisfies $\left(\alpha ,\alpha /2\sigma^{2}\right)$-RDP.*

**Lemma 1**  (Conversion from RDP to DP [30]). *Let f:D→R be a randomized mechanism that satisfies (α,ε)-RDP with α>1. Then, for any δ∈(0,1), f satisfies $\left(\varepsilon^{\prime },\delta \right)$-DP, where*

$$\varepsilon^{\prime }=\varepsilon +\frac{\log(1/\delta )}{\alpha -1}.$$

**Proposition 1**  (Divergency Accumulation [30]). *Let $f:D\to R_{1}$ be $\left(\alpha ,\epsilon_{1}\right)$-RDP and $g:R_{1}\times D\to R_{2}$ be $\left(\alpha ,\epsilon_{2}\right)$-RDP, then the mechanism defined as (X,Y), where X←f(D) and Y←g(X,D), satisfies $\left(\alpha ,\epsilon_{1}+\epsilon_{2}\right)$ -RDP.*

Proposition 1 provides a way to estimate the privacy budget when the noise multiplier varies during training. Our algorithm’s proof is derived from Proposition 1 and the definitions above.

## 4. Methodology

Before detailing the mathematical derivations, we provide a conceptual overview of SDD-DPSGD. For a precise definition of the variables and parameters used in our formulation, please refer to Table 1. Training a neural network on imbalanced data involves two distinct phases: an initial rapid “fitting” phase where foundational features are learned, and a later “compression” phase where the model fine-tunes. Standard DP-SGD applies constant noise, which often drowns out the weak signals of minority classes during the critical first phase. SDD-DPSGD solves this by mimicking a curriculum. We start with a high clipping threshold and low noise to protect fragile minority gradients, allowing the model to establish unbiased features. As training progresses, we exponentially decay the clipping threshold (to track naturally shrinking gradients) and exponentially increase the noise (to guarantee privacy), while lengthening the training steps to balance the privacy budget. The computational overhead of updating these scalars is O(1) per epoch, adding negligible cost compared to standard DP-SGD.

### 4.1. Noise and Multiplier Decay Function

#### 4.1.1. Theoretical Motivation: Unified Dynamics

To reduce hyperparameter complexity and avoid ad hoc tuning, we derive the relationship between the step size growth and noise multiplier based on the principle of Uniform Privacy Expenditure. We assume that each training stage should contribute equally to the overall privacy budget to prevent any single phase from dominating the RDP consumption. Approximating the RDP cost of stage *i* proportional to $D_{i}/\sigma_{i}^{2}$, the condition for uniform expenditure is $D_{i+1}/\sigma_{i+1}^{2}=D_{i}/\sigma_{i}^{2}$. Let λ be the dynamics factor governing the expansion of training steps, such that $D_{i+1}=\lambda \cdot D_{i}$ (where λ=1/γ). It follows that the optimal noise scaling must satisfy

$$\sigma_{i+1}=\sigma_{i}\cdot \sqrt{\lambda }.$$

This formulation unifies the step increment (γ) and noise ratio (β) into a single theoretical driver. Furthermore, the number of steps *n* is not arbitrary. It is constrained by the initial protection horizon. Furthermore, we define a parameter ρ as the Protection Constant for minimum initial phase fraction as in Table 1. To ensure the first phase $D_{0}$ is long enough to learn minority features, we require $D_{0}\geq \rho T$ (where ρ≈0.3). Under a geometric schedule determined by λ, $n=\lfloor \log_{\lambda }\left(1+\frac{\lambda -1}{\rho }\right)\rfloor$. This explains why a smaller *n* (e.g., n=3) is theoretically preferred over larger *n* which would fragment the critical early phase. The derivation is as follows.

Derivation of the Total Number of Steps *n*: To ensure that the model has a sufficient training duration to learn features from minority classes during the critical initial phase, we define a Protection Horizon Constraint ρ. Specifically, the duration of the first stage $D_{0}$ must account for at least a fixed proportion ρ of the total training budget *T*

$$D_{0}\geq \rho T.$$

In our SDD-DPSGD framework, the duration of each subsequent stage $D_{i}$ follows a geometric progression governed by the dynamics factor λ, such that $D_{i}=D_{0}\lambda^{i}$. For a schedule consisting of n+1 stages (from $D_{0}$ to $D_{n}$), the total training time *T* is the sum of this geometric series

$$T=\sum_{i=0}^{n}D_{i}=D_{0}\sum_{i=0}^{n}\lambda^{i}=D_{0}\frac{\lambda^{n+1}-1}{\lambda -1}.$$

Substituting the constraint $T\leq \frac{D_{0}}{\rho }$ into the summation formula, we obtain the following inequality

$$\frac{\lambda^{n+1}-1}{\lambda -1}\leq \frac{1}{\rho }.$$

To determine the maximum allowable number of scheduling steps *n*, we solve for *n* by applying the logarithm with base λ

$$\begin{array}{cc} \lambda^{n+1} & \leq 1+\frac{\lambda -1}{\rho }\\ n+1 & \leq \log_{\lambda }\left(1+\frac{\lambda -1}{\rho }\right) \end{array}$$

To maximize the granularity of the step-wise updates while strictly adhering to the protection requirement, we define the optimal *n* as:

$$n=\left\lfloor \log_{\lambda }\left(1+\frac{\lambda -1}{\rho }\right)\right\rfloor -1$$

This derivation formally links the scheduling complexity *n* to the dynamics factor λ and the protection threshold ρ, ensuring the theoretical consistency of our step-wise dual dynamic approach.

#### 4.1.2. Algorithm Implementation

Based on this theory, we calculate noise and clipping thresholds for every step $D_{i}$. Algorithm 1 details this estimation.
**Algorithm 1** sigma_clip_estimation**Input:** overall training epochs T, current epoch t, Step Increment Parameter γ, σ Noise Step Ratio β, clipping threshold decay parameter *a*, number of steps *n*, final noise multiplier $\sigma_{0}$, final clipping threshold $C_{0}$.
**Output:** current noise multiplier and clipping threshold $\sigma_{t},$$C_{t}$

  1: Divide T into multiple steps $D_{0}\ldots D_{n}$, where $\sum_{i}^{n}D_{i}=T$ and $D_{i}=D_{i+1}\cdot \gamma$

  2: **for**
*i* in {0,1,…,n−1} **do**

  3:     $\sigma_{i+1}=\frac{\sigma_{i}}{\beta }$

  4:     $C_{i+1}=C_{i}\cdot a$

  5: **end for**

  6: **if** t in $D_{i}$ **then**

  7:     $\sigma_{t}=\sigma_{i}$

  8:     $C_{t}=C_{i}$

  9: **end if**

10: **return** $\sigma_{t},C_{t}$


The key parameters governing the step-wise scheduling, including the decay rate *a* and noise ratio β, are formally defined in the notation summary (Table 1).

The step-wise scheduling can be explicitly formulated. Given an initial clipping threshold $C_{0}$ and initial noise multiplier $\sigma_{0}$, for any step i∈{0,1,...,n−1}, the parameters are updated via closed-form exponential schedules:

$$C_{i}=C_{0}\cdot a^{i}$$

$$\sigma_{i}=\frac{\sigma_{0}}{\beta^{i}}$$

where a∈(0,1) is the clipping decay rate and β∈(0,1) is the noise step ratio. Because β<1, the noise multiplier $\sigma_{i}$ exponentially increases to strictly bound the privacy leakage in later stages, while $C_{i}$ decays to prevent excessive noise injection ($\propto \sigma_{i}^{2}C_{i}^{2}$).

In our experiments, we adopt a conservative strategy for privacy safety. We set γ=0.9 (implying λ≈1.11) and β=0.8. Note that our noise growth (1/β=1.25) is strictly larger than the lower bound ($\frac{1}{\gamma }=\sqrt{\lambda }\approx 1.05$), providing an additional safety margin for late-stage training stability.

The relationship between $C_{t}$ and $C_{t+1}$, and $\sigma_{t}$ and $\sigma_{t+1}$ are implemented as $C_{t+1}=C_{t}\cdot a$ and $\sigma_{t+1}=\frac{\sigma_{t}}{\beta }$.

### 4.2. SDD-DPSGD

Algorithm 2 details our SDD-DPSGD method, which uses dynamic schedules for the noise multiplier and clipping threshold. The model, initialized with pretrained weights, trains for *T* iterations. In each iteration *t*, the algorithm first calls the sigma_clip_estimation function (Algorithm 1) to determine the current clipping threshold $C_{t}$ and noise multiplier $\sigma_{t}$. It then computes per-sample gradients, performs clipping using $C_{t}$, adds noise scaled by $\sigma_{t}$, and updates the model. The sampling probability for each batch *B* is q=B/|trainingDataset|.
**Algorithm 2** SDD-DPSGD**Input:** training datasets $\left\{x_{1},x_{2},\ldots ,x_{N}\right\}$, loss function $L(\theta ,x_{i})$. Parameters: learning rate η, Batch size for training *B*, initial noise multiplier $\sigma_{0}$, initial clipping bound $C_{0}$, clipping decay parameter *a*, Dynamics Factor λ (controls the training pacing), Protection Constant ρ (min. fraction of initial phase, e.g., 0.3).
**Output:** the final trained model $w_{T}$

  1: Initialize Dynamics Parameters

  2: γ←1/λ

  3: $\beta \leftarrow \sqrt{\gamma }$

  4: $n\leftarrow \lfloor \log_{\lambda }\left(1+\frac{\lambda -1}{\rho }\right)\rfloor$

  5: **while** t<T
**do**

  6:       Randomly sample a batch $B_{t}$ with *B* batch size and with probability $\frac{|B_{t}|}{N}$;

  7:       $\sigma_{t},C_{t}=sigma\_clip\_estimation\left(T,t,\gamma ,\beta ,a,n,\sigma_{0},C_{0}\right)$

  8:       **for** $x_{i}\in B_{t}$ **do**

  9:             Compute $g_{t}\left(x_{i}\right)\leftarrow \nabla L\left(w_{t},x_{i}\right)$

10:             ${\bar{g}}_{t}\left(x_{i}\right)\leftarrow g_{t}\left(x_{i}\right)/\max\left(1,\frac{∥g_{t}\left(x_{i}\right){∥}_{2}}{C_{t}}\right)$

11:       **end for**

12:       ${\tilde{\bar{g}}}_{t}\leftarrow \frac{1}{|B_{t}|}\left(\sum_{x_{i}\in B_{t}}{\bar{g}}_{t}\left(x_{i}\right)+N\left(0,\sigma_{t}^{2}C_{t}^{2}\right)\right)$

13:       $w_{t}=w_{t-1}-\eta {\tilde{\bar{g}}}_{t}$

14: **end while**

15: **return**
$w_{T}$


#### 4.2.1. Motivations

Our motivation stems from the unique challenges of applying differential privacy to deep learning models being fine-tuned.

We initialize our model with pretrained weights, meaning the initial feature-fitting stage was completed on a large, public, non-sensitive dataset. Consequently, our fine-tuning process focuses on the second phase of deep learning dynamics: compressing the input into an efficient representation [31]. This phase presents a dual challenge: protecting privacy under DP while preserving the critical signals from the minority class.

The core problem, as noted in related work [1,32], is that optimization on imbalanced datasets is numerically dominated by the strong, consistent gradients from the majority class. The gradient signals from the minority class, while critically important for fairness and accuracy, are inherently exceptionally fragile and susceptible to being submerged by the noise added for privacy.

**Figure 1:**
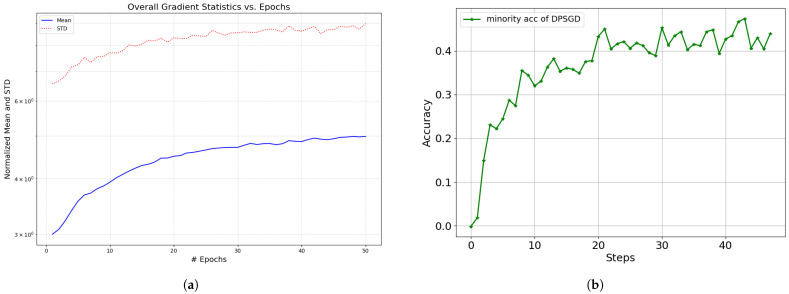
Motivation: gradient norms increase during training (**a**), while few-shot accuracy requires rapid fitting in early stages (**b**). (**a**) Mean and variance in gradients during differential private training using pretrained ResNet50 under ϵ=3,δ = $1\times 10^{-3}$. Symbol # Epoch on axis x stands for number of Epoch; (**b**) The accuracy curve of the few-shot group using DPSGD under conditions of ϵ=3,δ = $1\times 10^{-3}$.

This creates a dilemma for standard DPSGD, which uses a fixed clipping threshold *C* and noise scale σ. As shown in Figure 1a,b, gradient norms (both mean and std) tend to increase during the DP fine-tuning process.

(1) If we set a high, fixed *C*, the required noise ($\propto \sigma^{2}C^{2}$) becomes unacceptably large in the early stages, drowning the weak minority signals.

(2) If we set a low, fixed *C*, it will aggressively clip the informative late-stage gradients (biasing the model) and, more importantly, may clip the fragile but information-rich minority gradients early on. Therefore, our strategy is to dynamically schedule parameters to protect these crucial early-stage gradients. We begin the fine-tuning process with the following:

(1) A high clipping threshold ($C_{t}$) to ensure the direction and magnitude of fragile minority gradients are preserved.

(2) A low noise multiplier ($\sigma_{t}$) to maximize the Signal-to-Noise Ratio (SNR) when the model is learning the most foundational, generalizable features.

Crucially, our scheduling is governed by the principle of Uniform Privacy Expenditure. As training progresses, we increase the noise multiplier $\sigma_{t}$ to strictly limit the privacy leakage from late-stage gradients. To balance this increased noise, the training step duration $D_{t}$ must theoretically expand (i.e., $D_{t+1}>D_{t}$). This leads to our exponential step setting ($D_{i+1}=D_{i}\cdot \lambda$), which serves two purposes:

(1) Privacy Balance: Longer steps in later stages compensate for the higher noise levels, maintaining a constant rate of privacy budget consumption.

(2) Dynamics Matching: This pacing aligns with the fine-tuning dynamics in Figure 1b: a rapid initial “fitting” phase (protected by low noise) followed by a longer “compression” phase (stabilized by longer steps).

Finally, unlike standard decay schedules that might fragment the timeline, we impose an initial Protection Horizon Constraint. This ensures that the first step $D_{0}$ is sufficiently long to allow the optimizer to escape the initial biased saddle points before the noise level increases, effectively addressing the majority dominance issue noted in [32].

Theoretical Analysis of Cumulative Bias Reduction: In standard DP-SGD, the gradient is perturbed as ${\tilde{g}}_{t}={\bar{g}}_{t}+N\left(0,\sigma^{2}C^{2}\right)$, where ${\bar{g}}_{t}=g_{t}\min(1,C/∥g_{t}{∥}_{2})$. The clipping operation introduces a bias bounded by $∥E[{\bar{g}}_{t}]-\nabla L∥$. For a fixed clipping threshold *C*, if *C* is small, early-stage gradients (which have large norms) suffer from severe clipping bias. If *C* is large, the constant noise variance $\propto \sigma^{2}C^{2}$ prevents convergence. Our dual-schedule SDD-DPSGD explicitly reduces this cumulative bias. It is a well-established optimization phenomenon that the true gradient norm naturally decays as the model converges toward a local minimum. By defining the schedule $C_{i}=C_{0}\cdot a^{i}$, our clipping threshold dynamically tracks the natural decay of the gradient norms. This ensures that $P(∥g_{t}{∥}_{2}>C_{t})$ remains relatively small across all stages *i*, thereby maintaining $E[{\tilde{g}}_{t}]\approx \nabla L$ and significantly reducing the cumulative clipping bias over the entire training trajectory compared to static methods.

Convergence Guarantees and Variance Bound under the Schedule: Under standard non-convex DP-SGD assumptions (L-smoothness and bounded variance), the convergence error bound depends heavily on the injected noise variance at each step, which is proportional to $\sigma_{t}^{2}C_{t}^{2}$. In our step-wise dynamic schedule, at step *i*, the noise variance term is as follows.

$$\mathrm{Var}\left({\mathrm{Noise}}_{i}\right)\propto \sigma_{i}^{2}C_{i}^{2}={\left(\frac{\sigma_{0}}{\beta^{i}}\right)}^{2}{\left(C_{0}a^{i}\right)}^{2}=\sigma_{0}^{2}C_{0}^{2}{\left(\frac{a}{\beta }\right)}^{2i}.$$

Theoretical Justification for Gradient Preservation: To formally explain why this scheduling improves gradient preservation for minority classes, consider the clipping operation: ${\bar{g}}_{t}\left(x_{i}\right)=g_{t}\left(x_{i}\right)/\max\left(1,\frac{∥g_{t}\left(x_{i}\right){∥}_{2}}{C_{t}}\right)$. In the critical initial training phase ($t\in D_{0}$), our algorithm initializes with a deliberately large clipping threshold (e.g., $C_{0}=50$). Because the gradient norms of minority samples, while informative, are bounded, this large $C_{0}$ ensures that $∥g_{t}\left(x_{i}\right){∥}_{2}\leq C_{0}$ with high probability. Consequently, the denominator in the clipping operation becomes 1, resulting exactly in ${\bar{g}}_{t}\left(x_{i}\right)=g_{t}\left(x_{i}\right)$. This perfectly preserves both the magnitude and the direction $\frac{g_{t}\left(x_{i}\right)}{∥g_{t}\left(x_{i}\right){∥}_{2}}$ of the fragile minority gradients. As training progresses and the true gradient norms naturally decay, our decaying threshold $C_{t}=C_{0}\cdot a^{t}$ tightly tracks this decay, preventing excessive noise injection without aggressively truncating the converging signals. Crucially, based on our empirical validation (e.g., optimal a=0.6 and β=0.8), we establish that a<β. Consequently, the ratio (a/β)<1. This provides a strong theoretical guarantee: despite the noise multiplier $\sigma_{i}$ exponentially increasing to satisfy the Uniform Privacy Expenditure constraint, the actual absolute noise variance injected into the model updates strictly decays (or remains bounded) over time. Therefore, our schedule fully inherits the convergence guarantees of standard DP-SGD, with the added benefit that the optimization strictly contracts into a narrower minimum in the final stages. Furthermore, the formal derivation is as follows.

Formal Derivation of Decaying Effective Noise Variance: To quantify the impact of the privacy mechanism on optimization, we analyze the effective additive noise variance $V_{t}$ injected into the aggregate gradient at step *t*. In DP-SGD, the noise added to the clipped gradient sum follows $N(0,\sigma_{t}^{2}C_{t}^{2})$. Thus, the variance scale of the noise is defined as follow

$$V_{t}={\left(\sigma_{t}C_{t}\right)}^{2}.$$

Based on our proposed scheduling policy, the components evolve as follows: clipping threshold decay—$C_{t}=C_{0}\cdot a^{t}$, where a∈(0,1); noise multiplier growth—to satisfy the privacy expenditure constraints, the noise multiplier $\sigma_{t}$ increases exponentially, denoted as $\sigma_{t}=\sigma_{0}\cdot \beta^{-t}$, where β∈(0,1). Substituting these terms into the variance equation yields

$$V_{t}={\left(\sigma_{0}\beta^{-t}\cdot C_{0}a^{t}\right)}^{2}={\left(\sigma_{0}C_{0}\right)}^{2}\cdot {\left(\frac{a}{\beta }\right)}^{2t}.$$

By defining the ratio $\frac{a}{\beta }$ as the Noise-Decay Coefficient, and given our empirical validation that a<β (resulting in $\frac{a}{\beta }<1$), we establish the following asymptotic behavior

$$\lim_{t\to \infty }V_{t}=V_{0}\cdot \lim_{t\to \infty }{\left(\frac{a}{\beta }\right)}^{2t}=0.$$

This derivation formally proves that while the relative noise multiplier $\sigma_{t}$ grows, the absolute noise power $V_{t}$ vanishes geometrically. This ensures that the optimization preserves fragile minority gradients during the high-threshold initialization phase and subsequently contracts into a high-precision local minimum as the effective noise variance approaches zero.

#### 4.2.2. Details of the Algorithm

The core of the SDD-DPSGD algorithm lies in its noise and clipping threshold mechanisms. Unlike Auto-DPSGD-S and Auto-DPSGD-L, which start with the maximum noise multiplier, SDD-DPSGD begins with a low noise level to avoid sub-optimal solutions in the early training stages. Meanwhile, the clipping threshold starts high and gradually decays as training progresses, aligning with the typical training process. These initial settings help SDD-DPSGD outperform other variants, especially on imbalanced datasets.

Algorithm 2 is compatible with DP. The whole computations use per-sample gradients only and clip it before updates. We demonstrate the estimation of the total privacy budget should be as follows.

For every step with $\sigma =\sigma_{t}$ and clipping threshold equal to $C_{t}$, thus the sensitivity is considered to be $C_{t}$. Under these conditions, for step *t* the rdp is considered to be

$$rdp_{t}=D_{\alpha }\left(M\left(x\right)∥M\left(x^{\prime }\right)\right),$$

where

$$M^{\prime }\left(D\right)=M\left(D\right)+N\left(0,C_{t}^{2}\sigma_{t}^{2}\right).$$

**Theorem 1**  (N RDP Sequential Composition). *Let $D,D^{\prime }$ be adjacent datasets. Let $M_{1},\ldots ,M_{n}$ be a sequence of mechanisms where each $M_{i}:D\times R_{1}\times \cdots \times R_{i-1}\to R_{i}$ is $\left(\alpha ,\epsilon_{i}\right)$-RDP. Let $h_{n}\left(D\right)=\left(X_{1},\ldots ,X_{n}\right)$ be the composed mechanism, where $X_{i}=M_{i}\left(D,X_{1},\ldots ,X_{i-1}\right)$. Then $h_{n}$ is $\left(\alpha ,\sum_{i=1}^{n}\epsilon_{i}\right)$-RDP.*

**Proof.**  We prove this by mathematical induction on *n*.

Base Case (n=1): The mechanism is $h_{1}\left(D\right)=M_{1}\left(D\right)$. By definition, $M_{1}$ is $\left(\alpha ,\epsilon_{1}\right)$-RDP. The sum $\sum_{i=1}^{1}\epsilon_{i}=\epsilon_{1}$. Thus, the theorem holds for n=1.
Inductive Hypothesis: Assume the theorem holds for a composition of *k* mechanisms, where k≥1. Let $h_{k}\left(D\right)=\left(X_{1},\ldots ,X_{k}\right)$ be the *k*-fold composition. Our hypothesis is that $h_{k}$ is $\left(\alpha ,\epsilon_{1\ldots k}\right)$-RDP, where $\epsilon_{1\ldots k}=\sum_{i=1}^{k}\epsilon_{i}$.
Inductive Step: We must show that the theorem holds for n=k+1. The (k+1)-fold composition is $h_{k+1}\left(D\right)=\left(X_{1},\ldots ,X_{k},X_{k+1}\right)$. We can group the outputs as
  $$h_{k+1}\left(D\right)=\left(h_{k}\left(D\right),M_{k+1}\left(D,h_{k}\left(D\right)\right)\right).$$
  This has the exact form of a 2-mechanism composition, as provided in the problem description. Let $f=h_{k}$ and $g=M_{k+1}$. By the inductive hypothesis, *f* is $\left(\alpha ,\epsilon_{1..k}\right)$-RDP. By definition, *g* is $\left(\alpha ,\epsilon_{k+1}\right)$-RDP.

Let $Z=h_{k+1}\left(D\right)$ and $Z^{\prime }=h_{k+1}\left(D^{\prime }\right)$. Let $X=f\left(D\right)=h_{k}\left(D\right)$ and $X^{\prime }=f\left(D^{\prime }\right)=h_{k}\left(D^{\prime }\right)$. Let $Y\left(x,\cdot \right)=g\left(x,D\right)=M_{k+1}\left(D,x\right)$ and $Y^{\prime }\left(x,\cdot \right)=g\left(x,D^{\prime }\right)=M_{k+1}\left(D^{\prime },x\right)$. The joint densities are Z(x,y)=X(x)Y(x,y) and $Z^{\prime }\left(x,y\right)=X^{\prime }\left(x\right)Y^{\prime }\left(x,y\right)$.

Applying the 2-mechanism composition according to Proposition 1, we have

$$\begin{array}{cc} \exp\left[\left(\alpha -1\right)\;D_{\alpha }\left(h_{k+1}\left(D\right)\;∥\;h_{k+1}\left(D^{\prime }\right)\right)\right]\; & \leq \exp\left[\left(\alpha -1\right)\sum_{i=1}^{k+1}\epsilon_{i}\right]. \end{array}$$

Taking logarithm and dividing by (α−1) yields

$$D_{\alpha }\left(h_{k+1}\left(D\right)∥h_{k+1}\left(D^{\prime }\right)\right)\leq \sum_{i=1}^{k+1}\epsilon_{i}.$$

This completes the inductive step.

By the principle of mathematical induction, the theorem holds for all n≥1. □

**Remark 1.**  *By applying Theorem 1, the overall RDP for our algorithm is the sum over n steps*

$$rdp=\sum_{t=0}^{n}rdp_{t},$$

*where t is the step index. Therefore, our algorithm is (α,rdp)-RDP with α being the order of the Rényi divergence. We then convert this (α,rdp)-RDP guarantee to standard (ϵ,δ)-DP using Lemma 1*

$$\left(rdp+\frac{\log(1/\delta )}{\alpha -1},\delta \right)-\mathrm{DP}.$$

## 5. Experiments

We use HAM10000 and ISIC2019 lesion classification dataset to verify the effectiveness of the different algorithms. These two datasets are widely used benchmarks in dermatology, both of which share a critical real-world challenge: severe class imbalance. Furthermore, they represent different levels of complexity, with ISIC2019 being a larger and more challenging benchmark, allowing us to thoroughly test the robustness of our algorithm.

**Figure 2:**
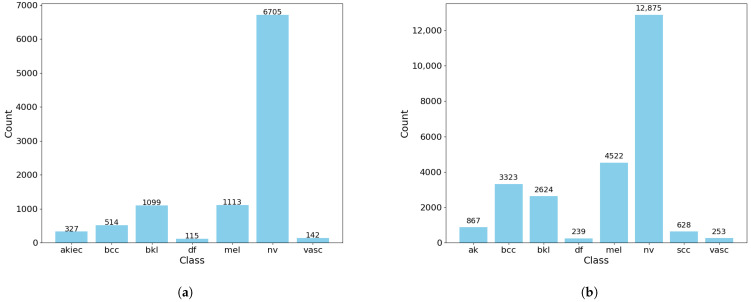
Distribution of the dataset. (**a**) Distribution of HAM10000 dataset. (**b**) Distribution of ISIC2019 dataset.

Without losing fairness, we chose to use RDP account for all the algorithm to test the effectiveness of the setting of different decay or noise step ratios. The implementation is available on GitHub (https://github.com/FangXieLab/SDD-DPSGD, accessed on 20 February 2026). HAM10000 is an imbalanced dataset dominated by one major class: Melanocytic Nevi (NV), and consists of 10,015 dermatoscopic images categorized into seven classes labeled from 0 to 6: Actinic keratoses (AK), Basal cell carcinoma (BCC), Benign keratosis-like lesions (BKL), Dermatofibroma (DF), Melanocytic nevi (NV), Vascular lesions (VASC), and Melanoma (MEL). The distribution of the overall dataset is presented as follows in Figure 2a.

Representing a more extensive and challenging benchmark, the ISIC 2019 dataset was released for the “Skin Lesion Analysis Towards Melanoma Detection” challenge and contains a larger training set of 25,331 images. It expands the multi-class classification task to eight categories, encompassing all seven classes from HAM10000 while introducing an additional class for Squamous Cell Carcinoma (SCC). Echoing the distributional properties of HAM10000, the ISIC 2019 dataset also exhibits a severe class imbalance, again dominated by the NV category. The increased scale and categorical diversity of the ISIC 2019 dataset provide a more complex and clinically realistic environment for developing and evaluating the robustness of automated diagnostic algorithms. The distribution of the overall dataset is presented as follows in Figure 2b.

Experimental Protocol and DP Implementation: For both HAM10000 and ISIC2019, images were resized to 224×224 and normalized using standard ImageNet statistics. We utilized an 80/20 train-validation split. The base architecture is a ResNet50 pretrained on ImageNet. Models were optimized using the SGD optimizer with a base learning rate of $1\times 10^{-4}$ and a batch size of 128. Training was conducted for a maximum of 50 epochs. Differential privacy was implemented using the Opacus library in PyTorch 2.6.0 +cu118. The schedule hyperparameters (γ,β,n) were derived analytically based on our theoretical framework, while the base learning rate was tuned non-privately prior to DP training (a common constraint in current DP literature). To ensure statistical significance, all reported metrics (Accuracy, MCC, F1, Recall) represent the mean over three independent runs, which is reflected in the variance analysis in our discussion.

Privacy Budget Selection and Convergence: We evaluate our models under three distinct privacy regimes: ϵ=3 (a strict privacy regime to test robustness against high noise), ϵ=8 (a standard moderate regime, widely accepted in industry applications), and ϵ=16 (a loose regime to demonstrate the algorithm’s upper-bound utility). Furthermore, as observed in our accuracy curves, training is intentionally halted before the curves completely flatten. In DP training, continuing for more epochs monotonically consumes the privacy budget. Stopping prior to full convergence ensures we do not violate the strict ϵ constraints.

### 5.1. Analysis of Parameter Dynamics

To validate the robustness of our theoretical framework, we analyze how the noise distribution responds to variations in our two core theoretical drivers: the dynamics factor (λ), which governs the pacing of privacy expenditure, and the Protection Constant (ρ), which constrains the initial phase duration. We focus on three key statistics: the initial noise value ($\sigma_{0}$), the weighted mean (μ), and the weighted variance.

#### 5.1.1. Impact of Dynamics Factor (λ)

**Figure 3:**
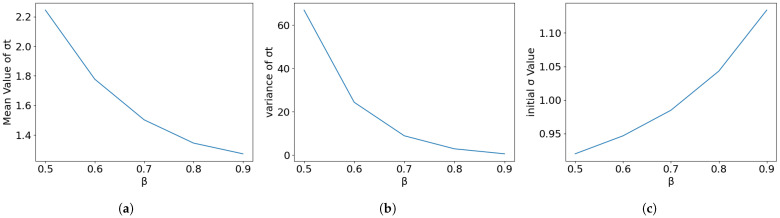
Dynamics coupling verification: Varying the noise ratio while holding dynamics fixed. The result supports a conservative choice (β=0.8) to minimize variance. (**a**) Mean of $\sigma_{t}$. (**b**) Variance in $\sigma_{t}$. (**c**) Initial $\sigma_{0}$.

The dynamics factor λ (where γ=1/λ) controls how aggressively the step size grows and the noise increases. Noise-Step Coupling Verification: Our theory suggests $\beta \approx \sqrt{1/\lambda }$. Figure 3 (varying β while fixing λ≈1.1) shows that deviations from this coupling create trade-offs. The intersection point around β=0.8 (slightly more conservative than the theoretical ≈0.95) offers a stability margin, reducing variance at the cost of a slightly higher initial $\sigma_{0}$.

**Figure 4:**
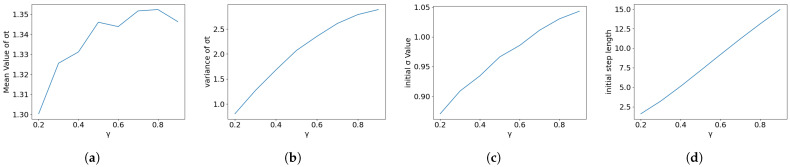
Impact of dynamics factor: Higher dynamics (lower γ) reduce initial noise (**c**) but dangerously compress the initial protection horizon $D_{0}$ (**d**). (**a**) Mean of $\sigma_{t}$. (**b**) Variance in $\sigma_{t}$. (**c**) Initial $\sigma_{0}$. (**d**) Initial Step $D_{0}$.

Dynamics Aggressiveness: Figure 4 illustrates the effect of varying the underlying dynamics (via γ). Stronger dynamics (lower γ, higher λ) significantly reduce the initial noise $\sigma_{0}$, theoretically aiding feature learning. However, the rightmost panel reveals the cost: aggressive dynamics exponentially compress the initial step length $D_{0}$. For instance, very high λ (low γ) reduces $D_{0}$ to negligible levels (≈2%), violating the protection horizon. This confirms that λ must be moderate (e.g., λ≈1.1, corresponding to γ=0.9).

#### 5.1.2. Impact of Protection Horizon Constraint (ρ)

**Figure 5:**
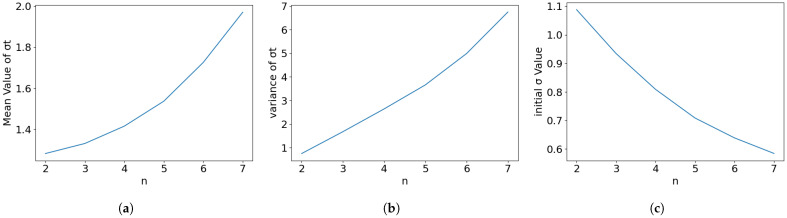
Validation of step derivation: Increasing steps beyond the derived optimal (n=3) destabilizes variance without significant noise reduction. (**a**) Mean of $\sigma_{t}$. (**b**) Variance in $\sigma_{t}$. (**c**) Initial $\sigma_{0}$.

The integer step count *n* is no longer a free parameter but is derived from λ and the required Protection Constant ρ (where $D_{0}\geq \rho T$). Figure 5 validates this constraint. Increasing *n* beyond the theoretically derived optimal (n=3) yields diminishing returns in reducing initial noise $\sigma_{0}$, while increasing overall variance. More critically, higher *n* fragments the schedule, reducing the effective ρ. This supports our derivation that given moderate dynamics (λ≈1.1), n=3 is the maximal solution that maintains a sufficient protection horizon (ρ≈0.3).

### 5.2. Empirical Validation of Parameter Theory

To validate the robustness of our theoretical framework proposed in Section 4.1.1, we conducted a sensitivity analysis on the HAM10000 dataset. Our objective shifts from merely searching for high-performing parameters to verifying whether the empirically optimal settings align with our derived theoretical constraints.

#### 5.2.1. Joint Validation of Noise Step Ratio (β) and Step Increment (γ)

Recall that our Uniform Privacy Expenditure theory suggests a coupling where $\beta \approx \sqrt{\gamma }$. We fixed n=3 and analyzed the interaction between these two parameters.

Step Increment (γ): Testing γ values (Table 2) revealed two local performance peaks at γ=0.3 and γ=0.9. However, consistent with our Initial Protection Horizon Constraint, γ=0.3 results in an excessively short initial phase $D_{0}$, which risks instability and fails to protect early minority gradients. The global optimum at γ=0.9 confirms that a more gradual expansion of training steps is necessary to maintain a sufficient $D_{0}$.

Noise Step Ratio (β): With γ fixed at 0.9, the theoretical neutral noise scaling would be $\beta_{\mathrm{theo}}\approx \sqrt{0.9}\approx 0.95$. As shown in Table 3, our empirical performance peaked at β=0.8. This value is lower than the theoretical neutral point 0.95, implying a noise growth factor of 1/0.8=1.25 (stronger than 1.05). This validates our conservative strategy: choosing a β slightly lower than the theoretical bound provides an additional stability margin, suppressing noise variance in later stages without compromising the initial signal.

#### 5.2.2. Validation of Step Count (*n*)

To understand the impact of schedule granularity, we tested n∈[3,5] (Table 4). The results show that n=3 yields the optimal accuracy. As *n* increases to 5, the performance drops. This empirically supports our derived constraint: increasing *n* fragments the timeline, reducing the initial phase $D_{0}$ below the critical threshold (ρ) required for learning minority features. Thus, n=3 is the maximal integer solution that respects the protection horizon.

#### 5.2.3. Empirical Selection of Clipping Decay Factor (*a*)

Instead of relying on heuristic estimations from non-private training, we determined the optimal clipping decay factor *a* through rigorous empirical evaluation. Fixing the initial clipping threshold at $C_{0}=50$, the noise multiplier at λ=1.1, and ρ=0.3, we performed a sensitivity analysis on *a*.

As detailed in Table 5, the model performance exhibits a clear concave trend. The accuracy improves as *a* increases from 0.4 to 0.6, reaching a peak of 80.63%. However, setting a=0.7 leads to a performance drop (79.02%). This indicates that a=0.6 offers the optimal decay rate, reducing the clipping threshold $C_{t}$ at a pace that best matches the model’s convergence needs without prematurely clipping informative gradients or retaining excessive noise.

### 5.3. Performance of Different Algorithms Within Different Privacy Setting

**Figure 6:**
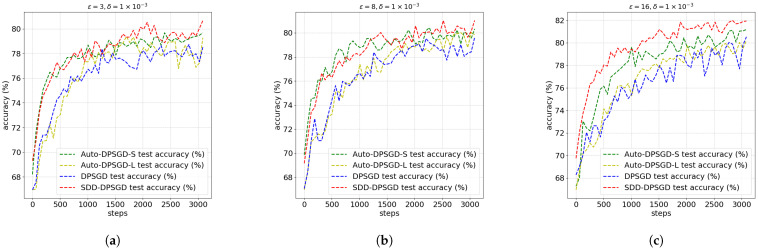
Performance of different algorithms on the HAM10000 dataset. The subfigures display the training curves under a fixed $\delta =1\times 10^{-3}$ across different privacy budgets: (**a**) ϵ=3; (**b**) ϵ=8; (**c**) ϵ=16.

Figure 6 and Table 6 and Table 7 summarize the performance of various algorithms on the HAM10000 dataset. We evaluate the models using standard classification metrics, including overall Accuracy, Matthews Correlation Coefficient (MCC) [33], Macro F1-score [34], and Recall [34]. MCC is particularly emphasized as it provides a more reliable statistical rate for severely imbalanced datasets.

We define the large-shot group as comprising one classes: NV. The few-shot group consists of AK, BCC, BKL, and MEL, DF and VASC. For ISIC2019, there is another class of SCC in the few shot group. The results show that SDD-DPSGD outperforms the other algorithms on the HAM10000 dataset, achieving a higher MCC, superior overall accuracy, and better performance on the few-shot group. To summarize, our algorithm enhances classification fairness, particularly for extremely imbalanced class distributions.

Furthermore, Table 8 and Table 9 present the corresponding results on the ISIC dataset. These findings are consistent with those observed on the HAM10000 dataset, further validating our conclusions and the efficacy of the proposed method.

## 6. Ablation Experiment

We conducted an ablation study to specifically validate the contribution of our dynamic privacy budget allocation mechanism. For this, we established a baseline using standard DPSGD equipped with the identical clipping mechanism of our SDD-DPSGD. The performance of this baseline, detailed in Table 10, serves as a direct point of comparison. The results clearly demonstrate that the dynamic allocation strategy itself yields substantial improvements in both overall model performance and the Matthews Correlation Coefficient.

## 7. Discussion

While standard DP-SGD mitigates privacy risks, it introduces a severe utility trade-off on imbalanced datasets, effectively acting as a disproportionate regularizer that penalizes underrepresented classes. Our SDD-DPSGD algorithm addresses this underlying optimization bottleneck by recognizing that deep learning relies on fragile, low-magnitude gradient signals in the early epochs to establish foundational feature representations.

Practical Implications for Hyperparameter Selection: A critical advantage of SDD-DPSGD for practitioners is that schedule hyperparameter selection does not require privacy-expensive validation. As demonstrated in our theoretical derivation (Section 4.1), the noise step ratio (β) and step increment (γ) are tightly coupled ($\beta \approx \sqrt{\gamma }$). By selecting a conservative dynamic factor (e.g., γ=0.9), practitioners can reliably set β=0.8 and the step count to n=3, leaving only the base learning rate for standard tuning.

Feasibility in Real-Time Applications: A critical consideration for medical AI is real-time applicability. It is important to distinguish between the training and inference phases; while SDD-DPSGD introduces complex scheduling during the offline training phase to satisfy DP constraints, it does not alter the underlying model architecture (e.g., ResNet50). Consequently, during the online inference phase, the model operates with standard computational complexity, making it highly realistic and efficient for real-time clinical diagnostic applications.

Generalizability and Limitations: Because the mechanism of SDD-DPSGD—protecting early-stage gradient directions from noise distortion—is mathematically domain-agnostic, we anticipate this approach will generalize well to other medical imaging modalities (such as radiology or pathology imaging) and non-medical imbalanced computer vision tasks. However, we acknowledge a limitation regarding its direct application to structured or tabular healthcare data. In tabular datasets, feature representations are not hierarchically learned over deep layers in the same manner as CNNs, and the gradient dynamics may differ. Extending dynamic DP scheduling to tree-based models or tabular neural networks remains an important subject for future investigation.

Clinical Impact and Minority Class Performance: While the overall accuracy improvements (e.g., ∼1%) may appear modest mathematically, overall accuracy is a highly misleading metric in severe class imbalance, as it is overwhelmingly dictated by the majority class (e.g., Melanocytic Nevi). The true practical significance of SDD-DPSGD is highlighted by its substantial gains in the Matthews Correlation Coefficient (MCC) and Recall, particularly in the “few-shot” groups. In real-world clinical deployments, such as federated hospital networks, failing to identify a minority class like Melanoma (false negatives) carries catastrophic patient risks. By preserving the fragile gradients of these rare lesions early in training, our dynamic schedule ensures the model does not sacrifice the safety of underrepresented patients for the sake of privacy compliance. This aligns with recent advances in privacy-aware learning frameworks for medical systems [35,36], highlighting the necessity of robust algorithms that do not marginalize rare conditions.

Future Work: To build upon our current findings, future research will proceed in two primary directions. First, addressing the growing demand for privacy in distributed environments, we plan to integrate the SDD-DPSGD algorithm into Federated Learning (FL) frameworks. This will involve evaluating its robustness against inference and adversarial attacks within resource-constrained settings. Second, although the HAM10000 and ISIC2019 datasets effectively demonstrate our algorithm’s capacity to mitigate severe class imbalance in dermatological image classification, extending this evaluation to a broader array of medical data modalities—such as 3D radiological scans and structured electronic health records—remains essential to fully establish its clinical generalizability.

## 8. Conclusions

In this work, we proposed SDD-DPSGD, a step-wise dual dynamic algorithm tailored for differentially private deep learning on class-imbalanced medical datasets. By implementing a high-to-low clipping threshold schedule alongside a low-to-high noise multiplier schedule, SDD-DPSGD effectively preserves critical minority-class gradients during the early stages of training. Extensive empirical evaluations on the HAM10000 and ISIC2019 datasets demonstrate that our method consistently outperforms existing dynamic DP-SGD baselines. Our method requires further adaptation for non-vision tasks like tabular healthcare data. Addressing these limits and exploring federated learning integration remain our future work.

## Figures and Tables

**Table 1:** Summary of primary mathematical symbols.

Symbol	Description
T,t	Overall training iterations (epochs) and current step.
N,B	Total dataset samples and batch size.
*q*	Sampling probability for each batch (B/N).
$B_{t}$	Randomly sampled batch at iteration *t*.
$w_{t},\theta$	Model weights/parameters at step *t*.
L	The loss function.
$g_{t}\left(x_{i}\right),{\bar{g}}_{t}\left(x_{i}\right),{\tilde{\bar{g}}}_{t}$	Per-sample, clipped, and noisy aggregated gradients.
ϵ,δ	Total privacy budget and relaxation bound.
$D,D^{\prime },x,x^{\prime }$	Neighboring datasets differing by one sample.
$\Delta_{k}f,s_{f}$	The $l_{k}$ norm sensitivity of a function *f*.
α	Order of the Rényi divergence.
$D_{\alpha }\left(A∥A^{\prime }\right)$	Rényi divergence of order α between two distributions.
$\sigma_{t},\sigma_{0}$	Injected Gaussian noise multiplier at step *t* and initial value.
$C_{t},C_{0}$	Gradient clipping threshold at step *t* and initial value.
$n,D_{i}$	Total scheduling stages and duration of the *i*-th stage.
γ∈(0,1)	The step increment parameter.
λ	Dynamics factor for training pacing (λ=1/γ).
β∈(0,1)	The noise step ratio.
ρ	Protection Constant for minimum initial phase fraction.
*a*	Clipping threshold decay parameter.

**Table 2:** Effects of step increment γ. Note that γ=0.9 achieves the best trade-off between stability and accuracy. (The bolded number stands for the highest accuracy).

γ	Accuracy (%)	γ	Accuracy (%)
0.3	80.63	0.7	80.38
0.4	80.18	0.8	79.68
0.5	79.88	0.9	**80.68**
0.6	78.63	1.0	79.68

**Table 3:** Effects of β (noise step ratio). The peak at β=0.8 confirms a conservative margin over the theoretical $\sqrt{\gamma }\approx 0.95$.

Parameter (β)	0.5	0.6	0.7	0.8	0.9	1
Accuracy (%)	78.84%	78.08%	79.03%	79.68%	79.18%	78.53%

**Table 4:** Effects of number of steps (*n*). Larger *n* fragments the initial phase, degrading performance.

*n* (Number of Steps)	n=3	n=4	n=5
Accuracy	80.63%	79.13%	78.98%

**Table 5:** Ablation study on clipping decay factor *a* (fixed parameters: $C_{0}=50,\lambda =1.11,\rho =0.3$).

Clipping Decay (*a*)	0.4	0.5	0.6	0.7
Accuracy (%)	79.18	79.83	**80.63**	79.02

**Table 6:** Overall performance (Accuracy, MCC, Macro F1, and Recall) of different algorithms on HAM10000 dataset with $\delta =1\times 10^{-3},\beta =0.8,\gamma =0.9,n=3$.

ϵ	Algorithm	Acc.	MCC	Macro F1	Recall
ϵ = 3	DPSGD	78.53%	56.60%	49.63%	47.09%
	Auto-DPSGD-S	79.68%	57.12%	48.54%	45.66%
	Auto-DPSGD-L	79.28%	57.72%	45.97%	43.98%
	SDD-DPSGD	**80.68%**	**59.83%**	**51.64%**	**47.40%**
ϵ = 8	DPSGD	79.63%	59.20%	52.75%	50.41%
	Auto-DPSGD-S	80.33%	61.24%	55.15%	**51.92%**
	Auto-DPSGD-L	80.03%	59.41%	50.27%	47.71%
	SDD-DPSGD	**81.03%**	**63.17%**	**55.96%**	51.59%
ϵ = 16	DPSGD	80.53%	60.80%	51.81%	49.35%
	Auto-DPSGD-S	81.18%	61.83%	49.77%	47.93%
	Auto-DPSGD-L	80.23%	61.83%	54.01%	51.09%
	SDD-DPSGD	**81.98%**	**64.51%**	**58.30%**	**55.55%**

**Table 7:** Average accuracy of different algorithms on large-shot, few-shot group of HAM10000 dataset with $\delta =1\times 10^{-3},\beta =0.8,\gamma =0.9,n=3$.

Algorithm	ε=3		ε=8		ε=16	
	Large Shot	Few Shot	Large Shot	Few Shot	Large Shot	Few Shot
DPSGD	94.1%	46.8%	95.9%	46.1%	96.1%	46.6%
Auto-DPSGD-L	96.3%	43.5%	94.6%	49.5%	94.4%	50.7%
Auto-DPSGD-S	96.3%	43.5%	94.6%	49.5%	94.4%	50.7%
SDD-DPSGD	96.6%	47.5%	95.4%	50.9%	96.0%	53.0%

**Table 8:** Overall performance (Accuracy, MCC, Macro F1, and Recall) of different algorithms on ISIC2019 dataset with $\delta =1\times 10^{-3},\beta =0.8,\gamma =0.9,n=3$.

ϵ	Algorithm	Acc.	MCC	Macro F1	Recall
ϵ = 3	DPSGD	67.00%	49.39%	31.39%	31.55%
	Auto-DPSGD-S	68.09%	50.76%	33.27%	33.14%
	Auto-DPSGD-L	67.55%	50.32%	31.36%	31.67%
	SDD-DPSGD	**69.11%**	**52.26%**	**35.81%**	**34.68%**
ϵ = 8	DPSGD	68.05%	50.97%	31.16%	31.60%
	Auto-DPSGD-S	69.43%	52.82%	33.71%	33.25%
	Auto-DPSGD-L	68.34%	51.71%	32.02%	32.35%
	SDD-DPSGD	**69.59%**	**53.62%**	**36.34%**	**35.28%**
ϵ = 16	DPSGD	68.60%	51.63%	32.56%	32.76%
	Auto-DPSGD-S	70.14%	53.97%	**36.68%**	34.80%
	Auto-DPSGD-L	68.32%	50.80%	32.17%	31.79%
	SDD-DPSGD	**70.34%**	**54.29%**	35.08%	**34.98%**

**Table 9:** Average accuracy of different algorithms on large-shot, few-shot group of ISIC2019 dataset with $\delta =1\times 10^{-3},\beta =0.8,\gamma =0.9,n=3$.

Algorithm	ε=3		ε=8		ε=16	
	Large Shot	Few Shot	Large Shot	Few Shot	Large Shot	Few Shot
DPSGD	90.5%	42.7%	92.2%	43.2%	92.4%	44.0%
Auto-DPSGD-S	92.7%	42.7%	92.3%	44.0%	92.0%	43.8%
Auto-DPSGD-L	93.0%	43.2%	92.4%	45.7%	92.5%	47.0%
SDD-DPSGD	92.7%	44.9%	91.3%	47.2%	91.9%	48.0%

**Table 10:** Comparison of accuracy and MCC of DPSGD and SDD-DPSGD under same clipping setting.

ϵ	Algorithm	Acc.	MCC	Macro F1	Recall
ϵ = 3	DPSGD	79.63%	58.47%	49.47%	46.29%
	SDD-DPSGD	**80.68%**	**59.83%**	**51.64%**	**47.40%**
ϵ = 8	DPSGD	80.78%	61.43%	50.90%	49.28%
	SDD-DPSGD	**81.03%**	**63.17%**	**55.96%**	**51.59%**
ϵ = 16	DPSGD	80.58%	60.81%	53.40%	50.06%
	SDD-DPSGD	**81.98%**	**64.51%**	**58.30%**	**55.55%**

## Data Availability

The implementation is available on GitHub https://github.com/FangXieLab/SDD-DPSGD, accessed on 20 February 2026. Public datasets (HAM10000 and ISIC2019) were used in this study.
